# Health and lifestyle advisors in support of primary care: An evaluation of an innovative pilot service in a region of high health inequality

**DOI:** 10.1371/journal.pone.0298955

**Published:** 2024-04-05

**Authors:** Lee Ingle, Rachel Martindale, Boluwatife Salami, Funsho Irete Fakorede, Kate Harvey, Sarah Capes, Grant Abt, Sarah Chipperfield

**Affiliations:** 1 School of Sport, Exercise and Rehabilitation Science, Faculty of Health Sciences, University of Hull, Hull, United Kingdom; 2 The Health and Wellbeing Service - Primary Care, City Health Care Partnership CIC, Marfleet Lane Surgery, Hull, United Kingdom; Osun State University, NIGERIA

## Abstract

**Introduction:**

A health and lifestyle advisor service embedded within primary care was piloted in Kingston-upon-Hull from January 2021. We aimed to evaluate the first two years of service delivery by identifying patient demographics referred to the service, reason for referral, determine uptake and retention rates, and monitor individual lifestyle-related risk factor changes following discharge.

**Methods:**

Anonymised data were extracted from the SystmOne database for all patients referred to the service between January 2021 and January 2023.

**Results:**

In the initial two years of the service, 705 unique patients were referred at a mean rate of ∼29 per month. Each unique patient received a median (robust median absolute deviation; [MAD]) of 3 (Steel N, et al 2018) planned consultations prior to discharge over this period. The majority of referrals were for symptom management and health promotion purposes (95%). Of those referred, 69% attended their appointments, and 14% did not attend. The majority of referrals were white British (55%), however, the service did receive a substantial number of referrals from minority ethnic groups, with only 67% of referrals speaking English as their main language. Eighteen distinct languages were spoken. Most referrals were classified as class I obese (59.4%). Across initial and final appointments, median (robust MAD) systolic blood pressure was 130 (15) mmHg and 130 (15) mmHg, and median (robust MAD) waist circumference was 103.0 (13.3) cm and 101.0 (13.3) cm.

**Conclusion:**

The evaluation highlighted the demand for this service embedded within primary care settings in Kingston-upon-Hull. Service engagement was evident, and a large proportion of those who engaged were from minority ethnic groups. A high proportion of referrals presented with obesity and/or hypertension which requires further investigation.

## Introduction

In 2019, 89% of deaths in the United Kingdom (UK) were caused by non-communicable diseases (NCDs), with individuals from more socio-economically deprived communities more likely to be affected [1, 2]. NCDs, often referred to as ’lifestyle-related’ or ‘chronic’ diseases manifest slowly over a long period and are largely preventable, requiring a long-term and systematic approach to treatment [2]. The four main diseases that negatively impact mortality and morbidity are cardiovascular disease, type 2 diabetes, cancer, and chronic respiratory disease [3], with poor diet, physical inactivity, smoking, and harmful use of alcohol being major modifiable individual risk factors [2]. The impact of NCDs is set to soar on a global basis due to two demographic trends; a rise in the global population, and a rapidly ageing population [3]. As NCDs disproportionately affect older people, the incidence of these diseases is likely to increase causing higher levels of morbidity, mortality, and disability, posing significant challenges for the provision of health care.

In the UK, there is considerable variation in years of life lost (YLLs) due to premature mortality. In 2016, the Global Burden of Disease study showed that in England, age-standardised rates of estimated YLLs due to the four leading NCDs were around twice as high in the most socio-economically deprived local authorities (compared to the least) [4]. Kingston-upon-Hull (Hull) is a port city situated in North East England, with a population in excess of 250,000 people. Hull has the second highest Index of Multiple Deprivation (IMD) score, and one of the highest age-standardised rates of YLLs in England [4], indicating high levels of health inequality within the city. Compounding this issue, the Hull region is disproportionately affected by a chronic shortage of general practitioners (GPs) [5]. Hull had the highest number of patients per GP in the country (3,185 patients per FTE GP), which is 80% more than the Wirral which had the lowest number [1,776 patients per FTE GP]) [6]. The region is geographically isolated which makes it difficult to recruit and retain GPs, and the combination of a chronic shortage of staff and increasing pressure on general practice due to rising workloads mean that regional health inequalities are likely to be reinforced [6].

In 2019, the NHS Long Term Plan [7] outlined plans to prevent illness and address health inequalities by adopting a comprehensive model of personalised care [8]. The model incorporated ‘health-coaching’ as a targeted intervention to support patients with long-term physical conditions. Health-coaching is designed to help people modify lifestyle behaviours and to take responsibility for their own health [9]. In Hull, novel ways of delivering the NHS Long Term Plan agenda were required. In 2021, an innovative multi-disciplinary primary care pilot service was introduced. The health and lifestyle practitioner service represented a novel approach to healthcare by providing both face-to-face and remote support to local referrals who required ongoing counselling to help improve current lifestyle-related behaviours. The service was similar to a model initially piloted in North America for people with diabetes [10]. The programme adopted a flexible self-management approach and enabled interventions to be tailored to patient needs [10].

The health-coaching model is designed to improve physiological, behavioural, psychological, and social outcomes in patients with chronic disease [11], and significantly improved one or more of the behaviours of physical activity, nutritional intake choices, weight management, or medication adherence [12]. A recent meta-analysis showed that health coaching led to small but significant improvements in physical activity, eating behaviour, and managing stress but was not effective for smoking cessation [13]. Despite the growing body of evidence supporting health-coaching, we are not aware of the model being evaluated in the UK. In the context of high GP caseload and significant health inequalities in the city of Hull, the primary care health and lifestyle practitioner service was developed as a multi-disciplinary approach to provide health coaching to support lifestyle behaviour change in order to manage local referrals with a history of chronic disease. The aim of the evaluation, conducted between January 2021 and January 2023, was to identify patient demographics on referral to the service, determine uptake and retention rates, and descriptively monitor individual lifestyle-related risk factors including alcohol consumption, smoking status, blood pressure, body mass, and obesity whilst engaged within the service.

## Materials and methods

### Ethics approval

Approval for a service evaluation was granted by the Faculty of Health Sciences Ethics Committee at the University of Hull. The conduct of a service evaluation was also approved by City Healthcare Partnerships internal approvals process. The service evaluation was not classed as research and so individual participant consent was not required, nor was formal Health Research Authority ethics approval. Data extraction from SystmOne was conducted by a data custodian at City Healthcare Partnerships on the 6th January 2023 which included data collected over the initial two years of the service. At no point did authors have access to information that could identify individual participants during or after data collection. Anonymised datasets were securely transferred to the chief investigator at the University of Hull in password-protected files.

### Scope of regional service

The innovative primary care health and lifestyle advisor service was implemented in January 2021 by the City Health Care Partnership Community Interest Company in the Nexus Primary Care Network in Kingston-upon-Hull. This network consisted of 15 GP practices. A total of 83,706 patients are registered with these practices representing 27% of patients registered with GP practices in Hull CCG [14]. In February 2022, the Haxby Group left the primary care network and it changed to Venn Primary Care Network with a total of 50,531 patients registered with the practices in the network [14]. The Kingston-upon-Hull integrated care system (ICS) is nationally ranked as the fourth most socio-economically deprived based on the proportion of lower-layer super output areas (LSOAs) [15]. Hull has low levels of ethnic diversity with minority ethnic groups accounting for 5.8% of the population compared to 14.6% in England [16].

### Deployment of advisors and health-coaching approach

In January 2021, three full-time equivalent graduate-educated health and lifestyle practitioners were recruited to the pilot service to support local referrals. Requirements for the role was a degree or equivalent qualification in health and/or physical activity, experience of working with individuals using knowledge of behaviour change theory to modify lifestyle behaviours, and working as part of a multidisciplinary team and in partnership with provider agencies.

Referred patients were offered support for up to 12 months commencing with an initial screening and assessment session and followed by regular one-to-one support consultations (where required) with an advisor allowing progression to be monitored and reviewed.

Consultations were either face-to-face in a clinic setting or by telephone. As the service was initiated during the Covid-19 pandemic, social distancing requirements meant that patients initially received telephone consultations only. As lockdown restrictions eased, face-to-face contacts resumed, patients had the choice of face-to-face or telephone consultations. Face-to-face consultations were conducted in clinic rooms in the geographical area of the patient’s home address to promote accessibility. Consultations lasted approximately 60 minutes and follow-up appointments were arranged in conjunction with the patient on a needs basis. If further follow-up consultations were deemed not to be required, then patients could be discharged at any point.

A flexible approach to health-coaching was used by the advisors to ensure support was individualised with a focus on implementing positive lifestyle changes. Advisors used models of behaviour change theory based on personal choice, however, the transtheoretical model was widely used [17]. This model helped to conceptualise where patients were in terms of readiness to change, and the behaviour change technique of motivational interviewing was used to progress patients to readiness to action [18]. Motivational interviewing, an evidence-based technique involving asking open-ended questions, incorporates using reflection and motivational language [19]. As health-coaching is a comprehensive approach and focuses on the individual, advisors supported patients through the process of changing behaviours by assisting them to develop a vision and identifying core values to sustain change over time [20]. The widely used SMART framework for goal-setting was used to set goals that were specific, measurable, attainable, realistic, and timed, and designed to empower patients to identify and overcome barriers, improve confidence and initiate long-term behaviour change to achieve their vision [21]. To further support patients in modifying lifestyle behaviours, the advisors worked in partnership with local agencies, for example, the local social prescribing service, through onward referral and signposting.

### Referral pathway

Patients were referred to the service by members of the multi-disciplinary team. Eligible patients were aged >18 years and initially registered at a Nexus Primary Care Network GP practice, or subsequently a Venn Primary Care Network GP practice (after February 2022).

- Initial inclusion criteria for referral:
- Score >10 on the NHS health check which provides an estimate of individual cardiovascular risk;
- Pre-diabetes, type 1 or type 2 diabetes, newly diagnosed type 2 diabetes, or uncontrolled type 2 diabetes;
- Mild, moderate or severe chronic obstructive pulmonary disease;
- Established cardiovascular disease or diagnosed with cardiovascular risk factors including hypertension and hypercholesterolaemia.

In spring 2022, the referral criteria were revised to include:

- Pre-diabetes (HbA1c between 42-47mmol mol^-1^ within the past 3 months);
- Score >10 on the NHS health check and QRISK score >10% (with the past 3 months).

The NHS health check is a programme that aims to reduce chronic disease in people aged 40–74 years without existing cardiovascular disease, hypertension or diabetes [22]. It involves assessing individual risk using the QRISK2 tool [23]. The estimated risk is expressed as a percentage of developing cardiovascular disease over the next 10 years, with an individual score >10 eligible for referral.

### Patient screening /assessment and ongoing support

Rates of patient referral and uptake were recorded, including number of appointments scheduled, appointments cancelled, and the number of patients who did not attend (DNA).

Age, sex, ethnicity, and main language spoken was recorded. Routinely collected anthropometric variables included stature, body mass index, body mass, waist circumference, and obesity status. Waist circumference risk was categorised for men: low <94 cm, high 94–102 cm, and very high >102 cm; and for women: low <80 cm, high 80–88 cm and very high >88 cm [24].

Individual lifestyle-related risk factors including smoking status, dietary intake, and alcohol consumption were also recorded based on self-report. These factors were evaluated because they reflect the major modifiable risk factors defined by the World Health Organisation (WHO) [2].

Categories for smoking status included: never smoked; ex-smoker; 1–9 cigarettes per day; 10–19 cigarettes per day; 20–39 cigarettes per day, >40 cigarettes per day. Alcohol intake was categorised as above or below the recommended safe limit of 14 units per week for men and women [25].

### Statistical analysis

Interventions were individualised based on need; therefore, patient referrals/appointments varied substantially. All quantitative data deviated from the normal distribution as assessed using the Shapiro-Wilk test and QQ plots. As such, for routinely measured key health outcomes including blood pressure, body mass index, body mass, and waist circumference, median (robust MAD) values are reported. Given that no control group was used, we are unable to examine changes over time in any variable. As such, no inferential statistics were conducted, and our data are purely descriptive.

## Results

Between January 2021 and January 2023, 705 unique patients were referred at a mean rate of ∼29 per month (Table 1). Each unique patient received a median (robust MAD) of 3 (4) planned consultations prior to discharge over this period. The majority of referrals were for symptom management and health promotion purposes (94.6% of referrals). Over the two-year evaluation period, 1,806 individual appointments were scheduled. Of those referred, 69% attended, and 14% failed to attend their appointments.

**Table 1:** Primary reason for referral to service and appointment uptake.

Reasons for referral	Number (n = 705)	Percentage (%)
**Health promotion**	566	80.3
**Social/financial**	18	2.6
**Symptom management**	101	14.3
**Emotional/psychological support**	18	2.6
**Cancelled by patient**	107	5.9
**Cancelled by service**	49	2.7
**Did not attend (DNA)**	257	14.2
**Attended/completed**	1244	68.9

An equal group of males (50.6%) and females (49.4%) were referred (Table 2), and the majority of referrals were aged between 40–69 years (79.2%). The majority of referrals were white British (55%), however, the service did receive a significant number of referrals from minority ethnic groups, with only 66.8% of referrals speaking English as their first language.

**Table 2:** Demographic profile of referrals to the service.

Demographic Characteristics	Percentage (%)
**Sex**	
Female	49.4
Male	50.6
**Age group (years)**	
20–29	2.5
30–39	6.8
40–49	29.2
50–59	25.6
60–69	24.4
70–79	8.5
80+	1.6
**Ethnicity**	
White British	55.0
Mixed British	5.6
Black African	6.8
Other white ethnic group	8.2
Asian	1.7
Other Asian	3.8
Other Black	6.4
Other ethnic group	9.7
Other mixed background	0.1
**Main spoken language**	
English	66.8
Arabic	5.8
Polish	3.0
Kurdish	3.7
Swahili	0.7
Farsi	0.7
Welsh	1.1
Latvian	1.5
Oromo	0.8
Rundi	0.4
Urdu	0.3
Indo-Iranian	0.8
Italic/Latin	1.9
Other	2.0

Table 3 showed that 97% referrals received at least one blood pressure (BP) assessment. Between baseline and the final visit, there were 54 fewer individuals (8%) with stage 2 and 3 hypertension. There were 16 more people (2%) with normal BP values after the final visit. Median (robust MAD) values for systolic blood pressure at baseline and final visit were 130 (15) mmHg and 130 (15) mmHg. Median (robust MAD) values for diastolic blood pressure at baseline and final visit were 80 (12) mmHg and 80 (9) mmHg.

**Table 3:** Resting blood pressure classification across baseline and final visit.

Category (mmHg)	Baseline (n = 683)	Final visit (n = 683)
**Normal <120/80 mmHg**	179	195
**Elevated <129/80 mmHg**	102	109
**Stage I hypertension <139 / <89 mmHg**	182	213
**Stage II hypertension <179 / 90+ mmHg**	208	163
**Stage III hypertension >180 / >120 mmHg**	12	3

A number of anthropometric variables were recorded. Body mass index was recorded in 88% of referrals (Table 4). Of those referred and with a measured BMI, 2.9% fell within a healthy category. The majority of referrals were classified as class I obese (59.4%). At the final visit, 20 fewer people (3%) were classified as obese with 15 (2%) more people being classed as overweight. Median (robust MAD) BMI values across the baseline and final visit were 31 (7) kg·m^2^ and 31 (7) kg m^2^. Body mass was recorded in 94% of referrals. Median (robust MAD) body mass were 88.9 (21.4) kg and 87.8 (21.8) kg across baseline and final visit. Waist circumference was recorded in 23% of referrals. In these individuals, median (robust MAD) waist circumference were 103 (13) cm and 101 (13) cm for baseline and final visit.

**Table 4:** BMI categories.

Category (kg/m^2^)	Baseline visit	Final visit
**Healthy 18–5 to 24.9**	88	88
**Overweight 25 to 29.9**	155	170
**Obesity I 30 to <35**	165	152
**Obesity II 35 to <40**	102	96
**Obesity III 40 or higher**	90	89

Smoking history (69% of referrals) and alcohol intake (71% of referrals) were recorded based on self-report (Table 5). Approximately 31% of referrals were non-smokers and 47% reported that they had quit smoking. 86.6% of referrals had up to three appointments where self-reported smoking habits were reported.

**Table 5:** Smoking and alcohol intake of referrals.

Category	Baseline (n = 487)	Final (n = 487)
**Non-Smoker**	152	153
**Ex-Smoker**	227	232
**Light (1-9cigs/day)**	42	40
**Moderate (10-19cigs/day)**	38	34
**Heavy (20-39cigs/day)**	17	17
**Very Heavy (40+cigs/day)**	1	1
**Alcohol units**	Baseline (n = 504)	Final visit (n = 504)
**< 14**	469	460
**> 14**	35	44

The vast majority of referrals reported that they drank alcohol below the national safety guidelines at baseline (93% reporting < 14 units of alcohol per week), which fell to 91% of referrals at the final visit. 91.5% of referrals had up to three appointments where self-reported alcohol intake levels were reported.

Table 6 highlights the individualised approach taken to treating referrals within the service. The case studies highlight three separate individuals who were retained in the service between 6 weeks and 4 months.

**Table 6:** Case studies highlighting the journey’s of three referrals recruited to the service.

	Referral 1	Referral 2	Referral 3
**Age**	70	51	55
**Sex**	Male	Female	Female
**Referral reason**	Type 2 diabetes mellitus (HbA1c: 53mmol/L); coronary heart disease (angina pectoris/stents); hypertension; hypothyroidism, obesity.	Type 2 diabetes mellitus (HbA1c 74 mmol/L); obesity; excessive alcohol consumption; sedentarism; social isolation	Type 2 diabetes mellitus (HbA1c 66 mmol/L); chronic obstructive pulmonary disease; hypertension; arthritis; obesity; chronic pain; mobility issues; excessive alcohol consumption
**Assessment**	BMI: 36 Physical activity: gym 3–4 times per week & cycling.	BMI: 45 Physically inactive. Receiving support from addictions service and secondary mental health service.	BMI: 53 Heavy smoker. Complex needs. Not engaging with diabetes clinic, weight management service, pain management clinic, community occupational therapist or secondary mental health services. Not ready to change lifestyle—priorities were mental health and housing support,
**Actions**	Educated about healthy eating, stress management and alcohol consumption. Referred to weight management service but cancelled due to weight loss. Signposted to local Diabetes support group and Diabetes UK. Advised about engaging with physical activity.	Educated about healthy eating and physical activity. Self-monitoring—food & physical activity diary. Pedometer given Information and leaflets on diabetes, physical activity and weight loss. Referred to Living with Diabetes course. Referred to weight management service. Referred to Tigers Trust charity for walking football & social interaction.	Discussed at MDT meeting. Referred to housing support service Liaised with community social services to support care needs. Supported engagement with diabetes clinic, weight management service and pain management clinic.
**Outcome**	HbA1c: 36mmol/L. Weight loss: 9.3 kg. BMI: 32	HbA1C: 46 mmol/L Weight loss: 8.0 kg BMI: 41 Physical activity: 1 hour walking daily. Attending walking football. Awaiting place on Living with Diabetes course.	Attended diabetes clinic and pain management clinic Visit from housing officer Social services support received Assessment with mental health team scheduled Assessment with weight management team scheduled
**Time from assessment outcome**	4 months	7 weeks	6 weeks

## Discussion

Our evaluation of the health and lifestyle advisor role based within some primary care centres in Kingston-upon-Hull highlighted a number of issues. It was encouraging that minority ethnic groups engaged with the service as factors relating to access are significant barriers to equitable care [26]. Language is one such barrier but with English not being the main language for one-third of referrals participating in the service and 18 distinct languages spoken, it did not appear to be a major barrier for accessing the service. However, it does highlight the complexities and challenges of a service which needs to serve its local communities. These challenges are further compounded by specific ethnic communities, for example, black and south Asian communities regularly present with higher rates of type 2 diabetes, stroke and ischaemic heart disease compared to white Caucasians [27]. Engagement with the service by service-users was 71%, with appointment attendance rates being very similar to the national outpatient attendance of 77% in England in 2020–2021 [28]. This would appear to indicate that referrals valued the service. The vast majority of participants (87%) reported alcohol consumption within UK health limit guidelines (<14 units per week). This may be due to under-reporting of alcohol consumption, as 20% of the British population completely abstain from alcohol [29] but may also reflect a different relationship between minority ethnic groups and alcohol consumption.

A primary care model that we can use as a comparator to the health and lifestyle advisor service is the primary care (GP) exercise referral scheme (ERS) model which was initially adopted in the UK in the early 1990s. There are currently >100 ERS around the UK [30]. Systematic review and meta-analysis of ERS trials have consistently shown poor evidence for their effectiveness; Pavey and colleagues [31] reported weak evidence of an increase in participants who achieved 90–150 minutes of moderate-intensity physical activity per week at 6–12 months’ follow-up. Furthermore, there was no evidence to support the effectiveness of ERS for improving moderate/vigorous physical activity adoption, blood pressure, body composition, psychological well-being or health-related quality of life when compared to alternative models of delivery. However, some of the uncertainties regarding the clinical effectiveness of ERS have focused on its impact in people with medical conditions and whether ERS can positively modify risk factors including blood pressure [31]. Our evaluation of the primary care health and wellbeing advisor service in Hull is unable to determine if clinically meaningful changes have occurred over time, due to a lack of a control group being. Our descriptive data shows that patients are willing to use the service and that both uptake and retention are satisfactory, however, controlled trials are now needed to determine if the lifestyle advisor programme is superior to the ERS or similar schemes. Further, a health economics evaluation to determine cost effectiveness is also required.

This evaluation was not without limitations. We were unable to report data on individual-level socio-economic status of the service users, consequently, we are unable to comment on the socio-economic status of those individuals who engaged with the service. The service initiated during the Covid-19 pandemic and subsequent lockdown restrictions led to some routine data not being collected and recorded. Initially, consultations were conducted by telephone so variables such as BMI and waist circumference were not recorded. Physical activity and quality of life data were not recorded in sufficient quantities to report within the timescales evaluated. A health economics analysis of the lifestyle advisor service was beyond the scope of this evaluation; therefore, we cannot comment on cost-effectiveness. However, we can conclude that there is a high demand for this service within the region to support general practice and alleviate pressures on general practitioners.

In conclusion, this evaluation highlighted the demand for this service embedded within primary care settings in Kingston-upon-Hull. Service engagement was evident, and a large proportion of those who engaged were from minority ethnic groups. A high proportion of referrals presented with obesity and hypertension which requires further investigation and intervention.

## Supporting information

S1 File (PDF)

S1 Data (CSV)

S2 Data (CSV)

S3 Data (CSV)

S4 Data (CSV)

S5 Data (CSV)

S6 Data (CSV)
