# Supplementary material for: Health and lifestyle advisors in support of primary care: An evaluation of an innovative pilot service in a region of high health inequality
Source: PLoS One. 2024 Apr 5;19(4):e0298955. doi: 10.1371/journal.pone.0298955 (PMC10997055; doi:10.1371/journal.pone.0298955)
Supplement: S1 File — (PDF) [file pone.0298955.s001.pdf]

University of Hull
Hull, HU6 7RX
United Kingdom
T: +44 (0)1482 463336 | **E:** e.walker@hull.ac.uk
w: www.hull.ac.uk

PRIVATE AND CONFIDENTIAL
Professor Lee Ingle
Faculty of Health Sciences
University of Hull
Via email

23 October 2020

Dear Lee

REF FHS288 - An evaluation of a Primary Care Health and Lifestyle Practitioner Service

Thank you for your responses to the points raised by the Faculty of Health Sciences Research Ethics Committee.

Given the information you have provided I confirm approval by Chair's action.

Please refer to the Research Ethics Committee web page for reporting requirements in the event of any amendments to your study.

Should an Adverse Event need to be reported, please complete the Adverse Event Form and send it to the Research Ethics Committee FHS-ethicssubmissions@hull.ac.uk within 15 days of the Chief Investigator becoming aware of the event.

I wish you every success with your study.

Yours sincerely

Professor Liz Walker
Chair, FHS Research Ethics Committee

Liz Walker | Professor of Health and Social Work Research |
Faculty of Health Sciences
University of Hull
Hull, HU6 7RX, UK
www.hull.ac.uk
e.walker@hull.ac.uk | 01482 463336

@UniOfHull /UniversityOfHull universityofhull
